# Dermatological side effects of dipeptidyl Peptidase-4 inhibitors in diabetes management: a comprehensive review

**DOI:** 10.1186/s40842-024-00165-w

**Published:** 2024-03-25

**Authors:** Shirin Zaresharifi, Mahtab Niroomand, Sarina Borran, Sahar Dadkhahfar

**Affiliations:** 1 Skin Research Center, Shahid Beheshti University of Medical Sciences, Tehran, Iran; 2 Internal Medicine Department, Endocrinology Division, Shahid Beheshti University of Medical Science, Tehran, Iran; 3 Medical student at Shahid Beheshti University of Medical Sciences, Tehran, Iran

## Abstract

Dipeptidyl peptidase-4 (DPP-4) inhibitors are a class of drugs that enhance the incretin-insulin pathway and offer effective glycemic control in type 2 diabetes mellitus. However, these drugs may be associated with various dermatological side effects, ranging from mild to severe. This review article summarizes the current literature on the dermatological side effects of DPP-4 inhibitors, including bullous pemphigoid, severe cutaneous adverse drug reactions, fixed drug eruptions, and other mucocutaneous reactions. The review also discusses the possible mechanisms, risk factors, diagnosis, and management of these side effects. This review aims to increase the awareness and vigilance of healthcare providers in recognizing and managing the dermatological side effects of DPP-4 inhibitors and to emphasize the need for further research and surveillance to optimize diabetes care and patient safety.

## Introduction

Diabetes mellitus is a chronic endocrine disorder that affects insulin secretion and action. It is a major global health problem that has been increasing rapidly in the past decades. According to the World Health Organization (WHO) and the International Diabetes Federation (IDF), the prevalence of type 2 diabetes (T2D) worldwide has more than doubled since 1980, and diabetes was the direct cause of nearly 1.6 million deaths in 2019. The WHO projects that diabetes will be the seventh leading cause of death by 2030.

Several medications are available to treat diabetes, such as insulin, insulin sensitizers, and glucose absorption inhibitors [1]. However, these drugs have limitations and side effects, such as hypoglycemia, weight gain, and gastrointestinal disturbances. Another class of medications, that promote glucose excretion from urine, is called sodium-glucose cotransporter 2 inhibitors (SGLT2 inhibitors). These drugs have benefits such as lowering blood pressure, weight loss, reduced risk of heart failure, and improved kidney function, but they also increase the risk of genitourinary tract infections and diabetic ketoacidosis. Therefore, there is a need for new and effective therapies for diabetes.

One of the recent advances in diabetes treatment is the discovery of the incretin-insulin pathway, which involves two hormones: glucagon-like peptide-1 (GLP-1) and glucose-dependent insulinotropic polypeptide (GIP). These hormones are secreted by the intestines in response to food intake and stimulate insulin secretion from the pancreas in a glucose-dependent manner. Based on this knowledge, two new classes of drugs have been developed: GLP-1 receptor agonists (GLP-1 RAs) and DPP-4 inhibitors.

DPP-4 inhibitors are oral drugs that prevent the degradation of GLP-1 and GIP by inhibiting the enzyme DPP-4, which cleaves these hormones. By increasing the levels and activity of GLP-1 and GIP, DPP-4 inhibitors stimulate insulin secretion, inhibit glucagon secretion, and improve glucose control, without causing hypoglycemia [2, 3].

They also have other beneficial effects on glucose metabolism, lipid metabolism, appetite regulation, gut motility, and immune function. They also have anti-inflammatory properties that reduce cardiovascular risk, help preserve beta-cell function, and improve renal function [4].

DPP-4 inhibitors have shown promising results in other diseases such as Alzheimer’s disease, COVID-19, and cancer [5].

Although these drugs have shown positive outcomes in clinical trials and practice, they are not free of adverse effects. Some of the common side effects of DPP-4 inhibitors are upper respiratory tract infections, urinary tract infections, headaches, and nasopharyngitis. Moreover, this class of drugs has been associated with rare but serious complications, such as pancreatitis, pancreatic cancer, thyroid cancer, and sometimes severe allergic reactions [3, 4].

Skin is the largest organ of the human body and contains various receptors and enzymes that are involved in different physiological processes. Some of these molecules are also present in other organs and systems. Therefore, any systemic drug, including those used for diabetes treatment, can potentially cause unintended reactions in the skin. These adverse reactions have been thoroughly reviewed about insulin and other oral agents however the literature on the cutaneous adverse effects of DPP-4 inhibitors is scarce and mainly based on case reports or small case series, with bullous pemphigoid (BP) being the only exception that has been studied in RCTs and large cohort studies. Therefore, our review aims to provide a comprehensive overview of the dermatological side effects of DPP-4 inhibitors in patients with diabetes based on the current knowledge.

BP, pruritus, particularly associated with Alogliptin and Sitagliptin, fixed drug eruptions primarily linked to Vildagliptin and Sitagliptin, and occurrences of drug reaction with eosinophilia and systemic symptoms (DRESS) and other skin reactions such as angioedema and photosensitivity have been identified as the most prevalent cutaneous adverse reactions associated with DPP-4 inhibitors in the reviewed literature.

### Bullous pemphigoid

BP is the most common autoimmune blistering disorder that affects mainly elderly patients and manifests as widespread pruritic tense vesicles and bullae. It is characterized by an autoimmune response directed against hemidesmosomal antigens (BP180 and BP230) at the dermoepidermal junction. Although the exact pathogenesis of BP remains unknown, it has been associated with several conditions including neurological disease, inflammatory skin diseases, and drugs.

**Fig. 1:**
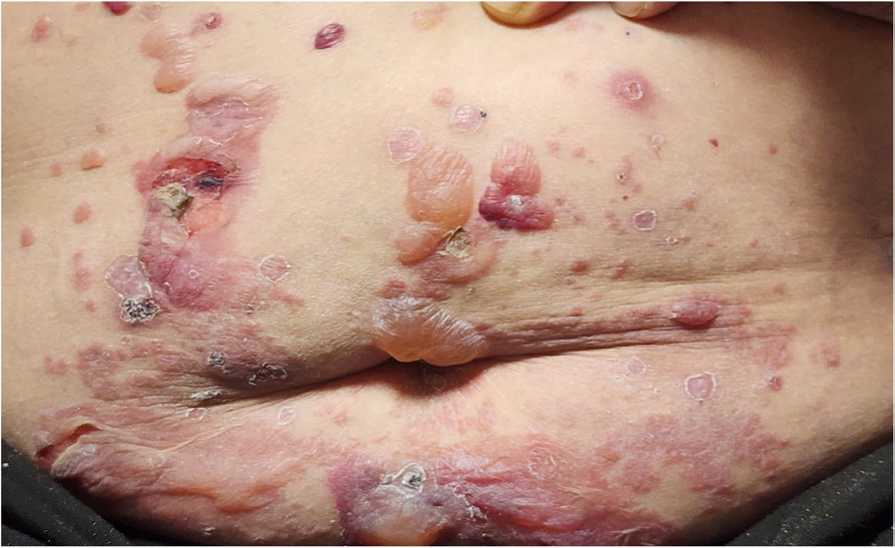
Bullous pemphigoid in a 73-year-old woman caused by linagliptin

A significant association between the use of DDP-4 inhibitors and the development of BP in patients has been observed in several large-scale studies (Fig. 1).

Based on these studies vildagliptin showed the strongest association with increased risk of BP among DDP-4 inhibitors, followed by sitagliptin and linagliptin [6].

Gender is not a predisposing factor for new-onset BP in DPP-4 inhibitor users. The specific timeframe between the initiation of gliptin agents and the manifestation of BP remains unclear and not well-established. Previous reviews have documented variable latency periods, with instances ranging from as short as 1 month to extending over 4 years. The mean latency period is reported to be from 6 to 19 months. The primary therapeutic approach involved discontinuing the use of the implicated drugs, followed by the administration of topical or oral corticosteroids, immunosuppressive medications, or intravenous immunoglobulin (IVIG), depending on the severity and extent of skin lesions. After the cessation of DPP-4 inhibitors, remission of symptoms typically occurred within a range of approximately one to 6 months [6–8].

Although BP induced by DPP-4 inhibitors has been extensively investigated in several studies, other dermatological adverse drug reactions (ADRs) associated with this class of drugs remain underexplored. We have compiled a summary of cutaneous ADRs induced by DPP inhibitors, other than BP, in two tables (Tables 1 and 2).

**Table 1:** Cutaneous reactions associated with dipeptidyl peptidase-4 inhibitors reported in RCTs, cohorts, and case series. (bullous pemphigoid not included)

No.	First author, year	Type of skin reaction	Culprit drug	Sample size	Prevalence	Comorbidities	Concomitant Medications
RCTs							
1.	Yosuke Takamiya,2019 [9]	Pruritus Non-specified skin rash	Teneligliptin	162	Pruritis & rash: 1% & 0.5% respectively	–	–
2.	M.A. Banerji,2010 [10]	Non-specified skin rash	Vildagliptin	2627	0.4%	Renal impairment	Metformin
3.	R E Pratley,2009 [11]	Pruritus	Alogliptin	585	1.5% & 3% in the Alogliptin 12.5 mg and 25 mg group respectively	–	–
4.	M A Nauck,2009 [12]	Pruritus, Eczematoid dermatitis	Alogliptin	420	Dry skin & Pruritis 1.2% Rash 2.1% Eczema 1.0%	–	Metformin
5.	Ralph A DeFronzo,2008 [13]	Pruritus	Alogliptin	420	12.5%	N/A	–
Cohort							
6.	Yuya Nakamura,2012 [14]	Exanthematous drug eruption	Alogliptin	16	6%	Renal failure	Hemodialysis
Case series							
7.	Shrey Desai,2010 [15]	SJS, TEN Angioedema, Anaphylaxis, Vasculitis	Sitagliptin	48	SJS, TEN:54% Angioedema: 9% Anaphylaxis: 31% Vasculitis:6%	–	–

(*N/A* not available: *SJS* Stevens-Johnson syndrome: *TEN* toxic epidermal necrolysis)

**Table 2:** Cutaneous reactions associated with dipeptidyl peptidase-4 inhibitors reported in case reports. (bullous pemphigoid not included)

No.	First author, year	Type of skin reaction	Culprit drug	Age/Sex	Comorbidities	Concomitant Medications	Time interval	Healing time after drug withdrawal
1.	Krupa A. Sunil,2022 [16]	Exanthematous drug eruption	Teneligliptin	63/M	Hypertension, Benign prostatic hypertrophy	Metformin, Glimepiride	A few hours	7 d
2.	Dana Al Masri,2021 [17]	FDE	Vildagliptin	58 /F	–	Metformin, Gliclazide, Empagliflozin	2 w	2 w
3.	Jessica Kaushal,2020 [18]	FDE	Vildagliptin	58 /M	–	Metformin	N/A	2 d
4.	Ankur Guliani,2019 [19]	Eczematoid dermatitis	Vildagliptin	54 /F	–	Metformin Voglibose	2 m	1 m
5.	Miao Zheng,2018 [20]	Exanthematous drug eruption	Linagliptin	41/F	Behcet disease	Prednisolone, Infliximab	2 m	1 m
6.	Elena Succurro,2017 [21]	Loss of eyebrows and eyelashes	Sitagliptin	69/M	–	Metformin	4 m	3 m
7.	Mrinal Gupta,2015 [22]	FDE	Sitagliptin	46/F	–	Metformin	1 w	5 d
8.	C Sin,2012 [23]	DRESS	Sitagliptin	66/F	Obesity, Hypertension	Metformin, insulin, Telmisartan	3 w	2 w
9.	Kaori Nakatani,2012 [24]	Exanthematous drug eruption	Sitagliptin	66/M	–	Metformin	6 m	2 m
10.	Sherea M Stricklin,2012 [25]	Photosensitivity reaction	Sitagliptin	65/ M	–	–	2 w	2 y
11.	Keiko TANAKA,2011 [26]	Exanthematous drug eruption	Sitagliptin	62/F	HTN	Trichlormethiazide, Telmisartan, Metformin, Glimepiride	2 w	1 w

(*M* male: *d* days: *FDE* Fixed drug eruption: *F* female: *w* weeks: *N/A* not available: *m* month: *DRESS* Drug Reaction with Eosinophilia and Systemic Symptoms)

### Severe cutaneous adverse drug reactions

Severe cutaneous adverse drug reactions (SCARs) are rare but potentially life-threatening conditions that involve extensive skin damage and systemic involvement. They include Stevens-Johnson syndrome (SJS), toxic epidermal necrolysis (TEN), DRESS syndrome, and anaphylaxis [27].

### SJS/TEN

Four cases of SJS and TEN have been documented, all of which were associated with sitagliptin use. The patients recovered without any fatalities. The demographic characteristics and the latency period of these cases were not reported [15].

### Anaphylaxis

In one case series 15 cases of anaphylaxis due to sitagliptin have been reported, with a mean age of 64 years (range: 38–84 years). The gender distribution was 6 males and 9 females. The average and median onset of symptoms from initiation of therapy was 8 and 5 days, respectively (range: 0–40 days). The majority of the patients (12 out of 15) received a dose of 100 mg of sitagliptin, while the remaining 3 had an unknown dose. More than half of the patients were hospitalized. The type of reaction included angioedema (5 out of 15), hypotension (1 out of 15), and no angioedema (9 out of 15). All of the cases had a resolution of the adverse reactions after discontinuation of sitagliptin [15].

### Dress

There is one case report of DRESS syndrome due to sitagliptin. In this case report a 66-year-old female patient with diabetes developed DRESS syndrome 3 weeks after starting sitagliptin therapy. She initially presented with a cutaneous reaction on her face and upper limbs, accompanied by a 40 °C fever, and later developed non-pruritic erythematous plaques on her inner thighs, mucosal ulcers, and renal failure. Her condition improved after discontinuing sitagliptin and receiving systemic corticosteroids for 2 weeks [23].

## Fixed drug eruptions

Three patients with diabetes (one male and two females aged 45 to 58 years old) reported symptoms of fixed drug eruption (FDE) after sitagliptin or vildagliptin was added to their diabetic medication. The lesions were cutaneous, mucosal, or both. Patients were started on DPP-4 inhibitors 1 week to 1 year before presentation. Symptoms resolved in 48 hours to a few weeks after discontinuation [21]. Surprisingly, two of these patients experienced poly sensitivity to different drugs. One patient who developed FDE on the leg after taking vildagliptin developed the same lesions to gliclazide, metformin, and empagliflozin too. The attributed common causative agent was magnesium stearate [17]. In the other patient, drug eruption occurred on the lips and the oral mucosa due to vildagliptin-metformin. Although the lesions subsided a few weeks after cessation of the drug, however, another episode of FDE happened in the exact location after taking ofloxacin-ornidazole. In this case, the suspected excipient was titanium dioxide nanoparticles [18]. As mentioned above in two out of three cases, the culprit was not the active ingredients of these medications but an excipient [17, 18, 22].

### Pruritus

Pruritus is the most common cutaneous ADR of DPP-4 inhibitors reported in RCTs, with alogliptin and teneligliptin being the most frequently implicated drugs. Interestingly, this side effect has been shown to increase with higher doses of alogliptin [9–13].

### Exanthematous drug eruption

Five cases of exanthematous drug eruption (EDE) have been reported in the literature, it was observed with alogliptin, sitagliptin, linagliptin, teneligliptin, and vildagliptin, time intervals varied from a few hours for teneligliptin to 6 months in sitagliptin, and mean and median healing time after drug withdrawal was 28 and 22 days respectively. The most common comorbidities associated with EDE were hypertension, obesity, and renal impairment, and the most common concomitant medication was metformin [14, 16, 20, 24, 26].

### Photosensitivity reactions

In one case, a patient exhibited persistent photosensitivity reactions characterized by excoriated edematous plaques on both forearms after receiving sitagliptin treatment. The diagnosis was conclusively confirmed through clinical and pathological assessments, with a notable improvement in the lesions observed 1 year after discontinuing the medication [25].

### Angioedema

DPP-4 inhibitors can extend the half-lives of bradykinin and substance P, therefore increasing the risk of hypersensitivity reactions, especially angioedema; which is among the hypersensitivity reactions reported in the prescribing information of most DPP-4 inhibitors as post-marketing events. Nineteen cases of angioedema have been reported by vildagliptin among which 73% were using ACE inhibitors concurrently, in one meta-analysis no significant increase in angioedema risk was seen with vildagliptin (without ACE inhibitors) compared to placebo or other oral hypoglycemic agents. Four cases of sitagliptin-induced angioedema have been also reported however no information about the concurrent use of other medications is available [28].

### Vasculitis

Only three cases of DPP-4 inhibitor-induced vasculitis have been reported, all of which were after taking sitagliptin. All three patients were on the highest dose of 100 mg and had a median onset of symptoms of 14 days after starting the therapy. The mean age of the vasculitis patients was 59 years, and there was one male and two female patients. None of them had any known comorbidities or concomitant medications. All three patients were hospitalized for their condition, and only one of them had a resolution of symptoms after drug withdrawal. The other two patients had unresolved vasculitis [15].

### Madarosis

In a unique case report, a 69-year-old male patient was noted to develop madarosis after 4 months of sitagliptin treatment [21].

## Conclusion

This manuscript provides a comprehensive review of the dermatological side effects associated with DPP-4 inhibitors; a class of medications used in diabetes management. While DPP-4 inhibitors offer effective glycemic control with lower hypoglycemic risk, this review highlights potential dermatological concerns, including SCARs like SJS and TEN, FDE, and an association with BP. Additionally, mucocutaneous reactions such as photosensitivity, pruritus, angioedema, vasculitis, and anaphylaxis have been reported. These findings underscore the importance of healthcare providers’ vigilance in recognizing and managing these dermatological side effects, emphasizing the need for ongoing research and awareness to optimize diabetes care and patient safety.

## Data Availability

Not applicable.
